# Supplementary material for: Catalase (CAT) Gene Family in Wheat (Triticum aestivum L.): Evolution, Expression Pattern and Function Analysis
Source: Int J Mol Sci. 2022 Jan 4;23(1):542. doi: 10.3390/ijms23010542 (PMC8745605; doi:10.3390/ijms23010542)
Supplement: Supplementary file 1 [file ijms-23-00542-s001.zip › Supplemental Figures.pdf]

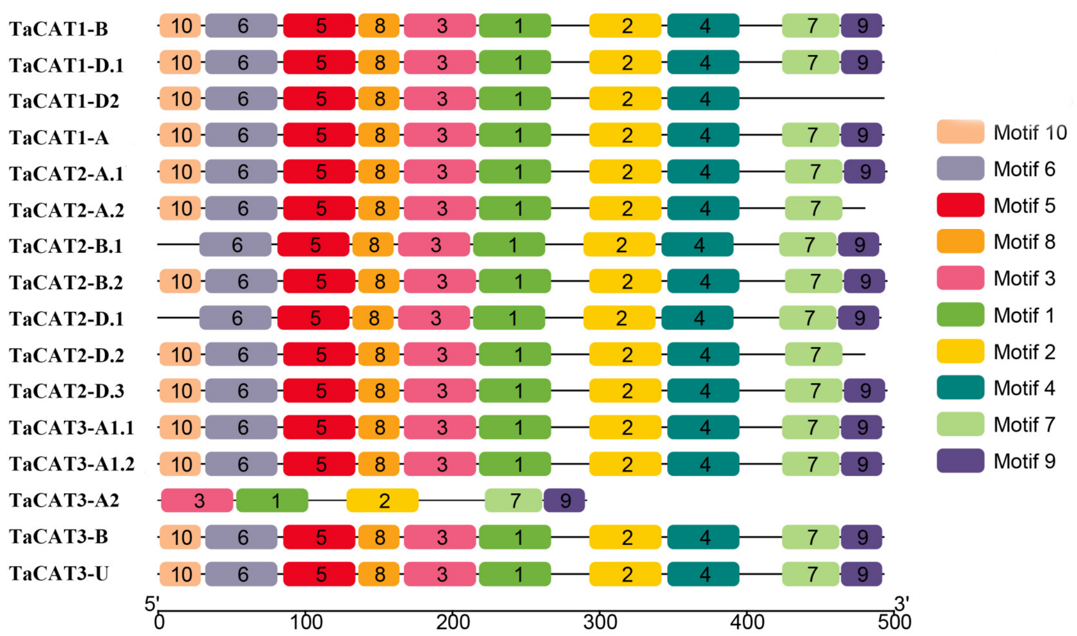

Figure S1. Distribution of conserved motifs in all TaCAT proteins. Using the protein sequences of TaCAT genes, Multiple Expectation maximization for Motif Elicitation (MEME) analysis was done to identify the conserved motifs in TaCAT proteins. Different motifs were indicated by different color boxes numbered 1-10. The multilevel consensus sequence of each motif was listed in Table S2.

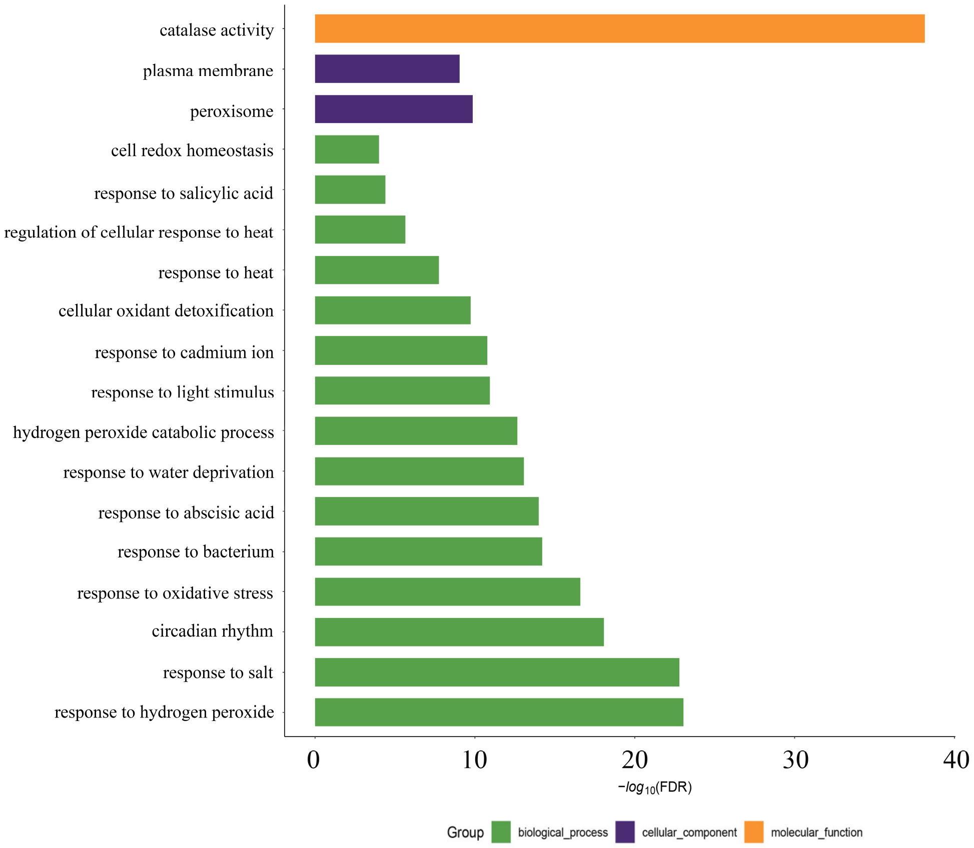

Figure S2. Gene Ontology (GO) enrichment analysis of the *TaCAT* genes. The green, purple, and orange columns represent the biological process (BP), molecular function (MF), and cellular component (CC) terms, respectively.

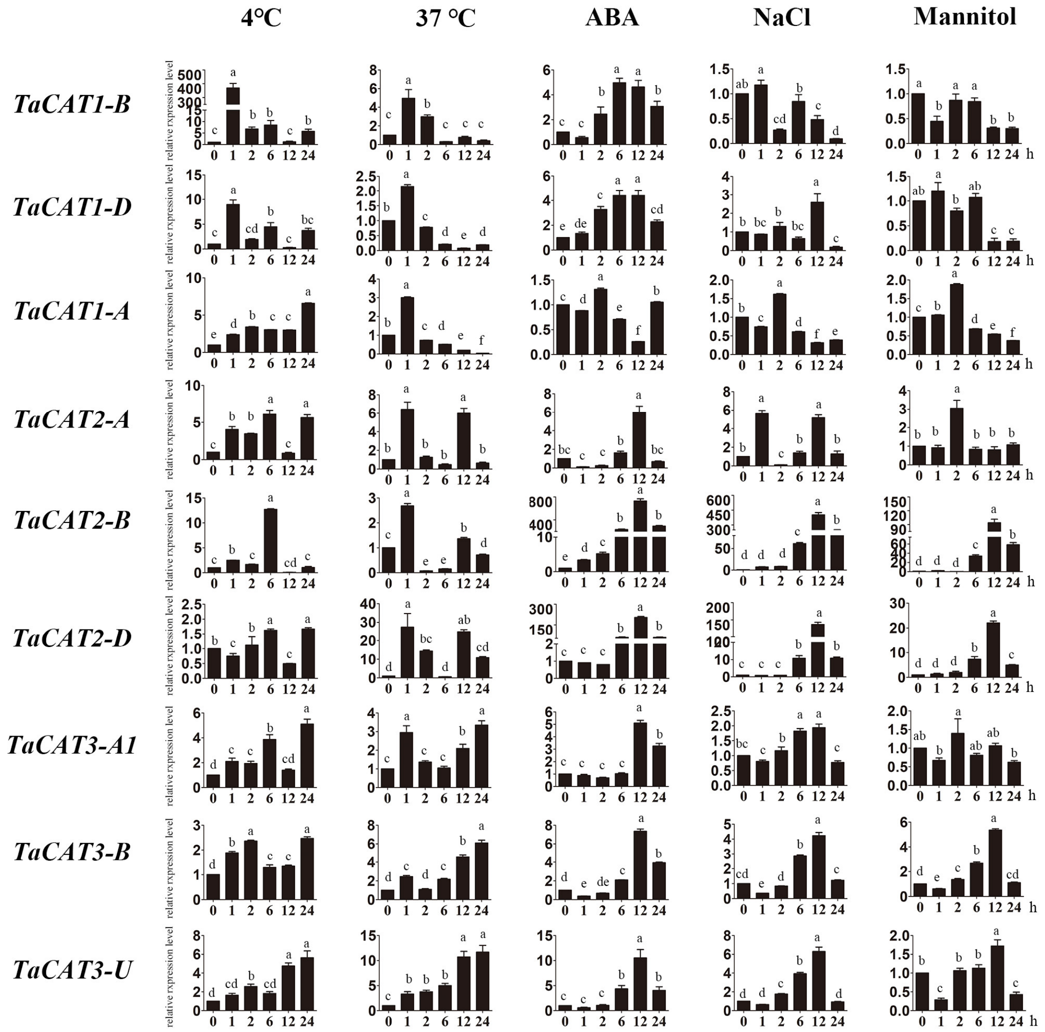

Figure S3. Expression analysis of *TaCAT* genes under different treatments by qRT-PCR. Relative expression levels of *TaCATs* in response to cold, heat, ABA, NaCl and mannitol treatments for 0 h, 1 h, 2 h, 6 h, 12 h and 24 h in the leaves at the three-leaf stage. Data were normalized with *GAPDH* gene, and ver-tical bars indicate standard deviation error. Different letters indicate significant differences at $P < 0.05$ according to one-way ANOVA and post-hoc Tukey's test.
